# Pharmacovigilance in practice: erythropoiesis-stimulating agents

**DOI:** 10.1002/cam4.275

**Published:** 2014-06-03

**Authors:** Michael Hedenus, Heinz Ludwig, David H Henry, Eduard Gasal

**Affiliations:** 1 Sundsvall HospitalSundsvall, Sweden; 2 WilhelminenspitalVienna, Austria; 3 Joan Karnell Cancer Center, Pennsylvania HospitalPhiladelphia, Pennsylvania; 4 Amgen Inc.Thousand Oaks, California

**Keywords:** Adverse event, chemotherapy induced anemia, erythropoiesis-stimulating agent, pharmacovigilance, safety signal

## Abstract

Pharmacovigilance (PV) is the science and activities relating to the detection, assessment, understanding, and prevention of adverse effects or other problems related to medical products after they have been licensed for marketing. The purpose of PV is to advance the safe use of marketed medical products. Regulatory agencies and license holders collaborate to collect data reported by health care providers, patients, and the public as well as data from systematic reviews, meta-analyses, and individual clinical and nonclinical studies. They validate and analyze the data to determine whether safety signals exist, and if warranted, develop an action plan to mitigate the identified risk. Erythropoiesis-stimulating agents (ESAs) provide an example of how PV is applied in reality. Among other approved indications, ESAs may be used to treat anemia in patients with chemotherapy-induced anemia. ESAs increase hemoglobin levels and reduce the need for transfusions; they are also associated with a known increased risk of thromboembolic events. Starting in 2003, emerging data suggested that ESAs might reduce survival. As a result of PV activities by regulatory agencies and license holders, labeling for ESAs addresses these risks. Meta-analyses and individual clinical studies have confirmed that ESAs increase the risk of thromboembolic events, but when used as indicated, ESAs have not been shown to have a significant effect on survival or disease progression. Ongoing safety studies will provide additional data in the coming years to further clarify the risks and benefits of ESAs.

## Introduction to Pharmacovigilance

A drug is approved if the risk/benefit balance is judged to be positive for the target population in the specified indication at the time of authorization 1. At that time, however, information on the safety of a medicinal product is limited due to many factors such as small numbers of patients in clinical trials; restricted population in terms of age, gender, and ethnicity; restricted comorbidity, comedications, and conditions of use; short duration of exposure and follow-up; and statistical problems associated with looking at multiple outcomes. Thus, not all actual or potential risks may be identified at the time an initial authorization is sought; many of the risks associated with the use of a medicinal product will only be discovered and characterized postauthorization with broader use.

**Figure 1:**
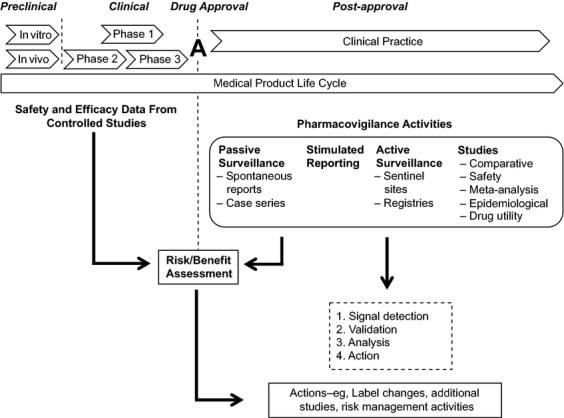
Pharmacovigilance overview. After a drug is approved for use in clinical practice, assessment of its risk and benefits is a continuous process based on new data obtained through various pharmacovigilance activities. License holders work closely with regulatory agencies to analyze these data and respond with appropriate action, which might include label changes, risk mitigation activities, and required additional (safety) studies to further characterize the drug.

Pharmacovigilance (PV) is defined as the science and activities relating to the detection, assessment, understanding, and prevention of adverse effects or any other problem related to medical products 2–4. Regulatory agencies such as the U.S. Food and Drug Administration (FDA) and the European Medicines Agency (EMA) provide guidance to marketing authorization (license) holders for PV efforts 2,3. These agencies may require license holders to establish and maintain PV activities; to acquire and analyze data about adverse events and adverse drug reactions reported spontaneously by health care professionals, patients, and the public (passive surveillance); and to monitor data from registries (active surveillance) and from comparative observational or targeted clinical investigations. The type of PV surveillance activity employed will be dependent on the frequency and the specificity of the given event (Table 1) 5. Further investigation may be warranted if there is identification of a potential safety signal, a concern about an excess of adverse events compared to what would be expected to be associated with a product's use 3. License holders then work with regulatory agencies to synthesize available information to continuously assess the risks and benefits, and determine the next steps (e.g., label changes, additional studies, risk management activities). Figure1 provides an overview of PV concepts and activities. Health care professionals play an important role by reporting adverse events or adverse drug reactions spontaneously whenever they are observed, whether or not they believe the event is associated with a particular drug. Procedures for reporting adverse events may vary by region and country.

**Table 1:** Strengths and limitations of different PV surveillance activities

PV surveillance activity	Strength	Limitation
Preclinical findings	• May identify possible adverse events early	• Needs confirmation from other PV surveillance activities
	• May provide a mechanism for an adverse event	
Spontaneous reporting	• Usually the first indication of a potential safety signal	• Cannot differentiate between new safety signals or deterioration of preexisting conditions
		• Reporting bias
Observational studies	• Allows rapid assessment of possible adverse events	• Data on very rare events may not be collected or observed in databases
	• Large sample size	• Causality may be unclear
		• Drugs are not always used according to their label
Clinical trials	• Prospectively tests for the presence of a potential safety signal	• Expensive
	• Provides the highest quality of data	• May take a long time to accumulate data
		• Rare events are difficult to detect due to small sample size
		• There may be conflicting results between individual trials due to varying trial design characteristics
Meta-analyses	• Summarizes data from multiple trials	• Aggregation of heterogeneous data may confound the interpretation of the results
	• Large sample size	

## Pharmacovigilance in Practice: Erythropoiesis-Stimulating Agents for Chemotherapy-Induced Anemia

Erythropoiesis-stimulating agents (ESAs) are indicated for use to treat anemia in patients with cancer receiving concomitant myelosuppressive chemotherapy. Successful treatment is characterized by increases in hemoglobin levels and elimination or reduction in red blood cell transfusion requirements; some studies have also shown reduction in fatigue and improvement in quality of life 6,7.

Two ESAs, epoetin alfa (EPOGEN, Amgen, Thousand Oaks, CA/PROCRIT, Janssen, Horsham, PA) and darbepoetin alfa (Aranesp, Amgen Inc.), are licensed in the United States for chemotherapy-induced anemia (CIA). Epoetin alfa (EPREX/ERYPO, Janssen), epoetin beta (NeoRecormonn, Roche, Welwyn Garden City, UK), epoetin theta (Eporatio, Ratiopharm, Ulm, Germany), and darbepoetin alfa (Aranesp, Amgen Inc.) are licensed in Europe for CIA. ESA biosimilars became available in Europe in 2007 (see below). The U.S. clinical and regulatory history of ESAs provides an example that demonstrates the importance of PV and the required close collaboration between license holders and regulators.

In addition to the known benefits of hemoglobin increases, transfusion reduction and possible reduction in fatigue, some early studies and meta-analyses showed a potential survival benefit and improved tumor response from use of ESAs in patients with cancer receiving chemotherapy 8,9. These results encouraged evaluation of ESAs in clinical studies in a wider range of indications, beyond those initially approved. The outcomes of these studies were unexpected. Starting in 2003, emerging results suggested adverse effects on survival and time to tumor progression in two large randomized controlled trials (BEST 10 and ENHANCE 11), leading to a review of ESAs by the FDA. In contrast to the studies supporting marketing approval and labeling claims, the BEST and ENHANCE studies were specifically designed to test whether use of an ESA to achieve and maintain high hemoglobin levels (12–15 g/dL) would improve tumor outcomes and survival compared to standard supportive care 11,12. Instead, BEST and ENHANCE showed evidence of detrimental survival and tumor outcomes. Additional epoetin alfa trials were terminated prematurely in 2003 because of unacceptable increases in the risk of thrombotic and cardiovascular events in the ESA arm (GOG 191 13, EPO-CAN-15 14, PR00-03-006 15) 16.

In May 2004, darbepoetin alfa label warnings and precautions were revised to include new information about thrombotic events, response rate, time to progression, and overall survival. In addition, the FDA and its oncologic drug advisory committee (ODAC) met in 2004 and agreed that future studies should be randomized, double-blind, and placebo-controlled, assessing survival in a homogeneous cancer subtype, to adequately assess the risks of ESAs in the approved indications. In 2005 and 2006, license holders (in the United States, Amgen Inc. and Johnson & Johnson, New Brunswick, NJ), in collaboration with the FDA, specified ongoing ESA studies in breast 17–19, cervical 20, and small-cell lung cancer 21 and non-Hodgkin lymphoma 22 as studies that should further clarify the risk of ESAs.

**Figure 2:**
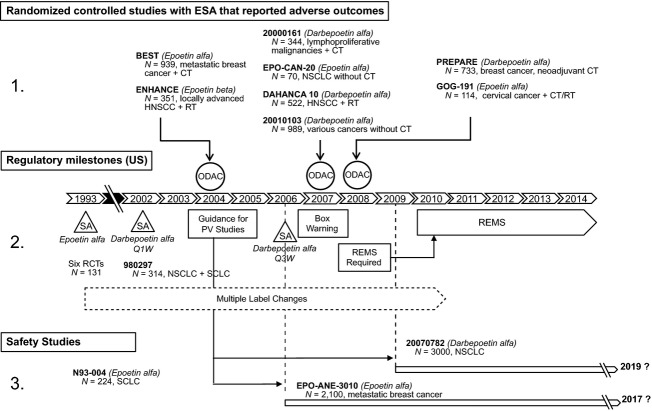
Regulatory history of ESAs in the United States. After approval for CIA, three FDA oncologic drug advisory meetings (ODACs) were conducted to review safety data from studies of ESAs used in patients with cancer. ESAs in cancer are currently indicated only for patients receiving concomitant chemotherapy; the U.S. label also specifies that ESAs should not be used when the anticipated treatment outcome is cure. CIA, chemotherapy-induced anemia; CT, chemotherapy; ESA, erythropoiesis-stimulating agent; FDA, Food and Drug Administration; HNSCC, head and neck squamous cell carcinoma; NSCLC, non–small-cell lung cancer; ODAC, oncology drug advisory committee; RCT, randomized controlled trial; RT, radiotherapy; SA, supplemental approval for chemotherapy-induced anemia; SCLC, small-cell lung cancer.

In May 2007, a second ODAC meeting was requested by the FDA after results of four additional studies became available showing adverse effects on survival and tumor progression 23. Study 20010103 was conducted in a heterogeneous population of patients with nonmyeloid malignancies not receiving chemotherapy 24. Study 20010161 was conducted in patients with lymphoproliferative malignancies receiving chemotherapy 25; adverse effects on survival had not been observed in an earlier dataset 26, but first became apparent after additional long-term follow-up was conducted. EPO-CAN-20 was a small quality-of-life study in patients with non–small-cell lung cancer (NSCLC) receiving radiotherapy without chemotherapy; the study was terminated prematurely 27. One additional study (DAHANCA 10) in patients with head and neck cancer receiving radiotherapy clearly showed an adverse effect on time to tumor progression 28. Based on the review of this new safety information, in March 2007 the FDA required license holders to revise product labeling to add a “boxed warning” describing increased risks of death, serious cardiovascular and thromboembolic events, and more rapid tumor progression. Clarification of dosing strategies was also required. One year later, a third ODAC meeting was convened by the FDA based on results of two new additional studies showing shorter survival and/or poorer loco-regional control 29. The first study, PREPARE, was an open-label, randomized study comparing the efficacy of two sequential neoadjuvant chemotherapy regimens in early breast cancer patients 19. An interim analysis was performed after a median follow-up of 3 years, at which time the survival rate was lower in the ESA treatment group. Additional analyses were reviewed from the second study (GOG-191) in patients with cervical cancer receiving radiochemotherapy, which had first been presented to the 2004 ODAC. Both local and distant recurrences were more frequent with ESAs; overall and progression-free survival were lower in the ESA arm versus control. In April 2008, based on the recommendation of the ODAC, the FDA required a Risk Evaluation and Mitigation Strategy (REMS) to ensure that the benefits of ESAs outweigh the risks; the REMS program was implemented in March 2010 in the United States only (Fig. 2). Table 2 summarizes the approved EMA and FDA labels for darbepoetin alfa for CIA as of September 2013.

**Table 2:** Approved indications for darbepoetin alfa in patients with CIA

	U.S. prescribing information 6	EU summary of product characteristics 7
Indication	Treatment of anemia due to the effects of concomitant myelosuppressive chemotherapy; upon initiation, there is a minimum of additional 2 months of planned chemotherapy	Treatment of symptomatic anemia in adult cancer patients with nonmyeloid malignancies receiving chemotherapy
Limitations of use	Aranesp has not been shown to improve quality of life, fatigue, or wellbeing.	Not applicable
	Not indicated for use:	
	• For patients with cancer receiving hormonal agents, biologic products, or radiotherapy, unless also receiving concomitant chemotherapy	
	• As a substitute for red blood cell transfusion for immediate correction of anemia	
Contraindication	• Uncontrolled hypertension	• Hypersensitivity to the active substance or to any of the excipients
	• Pure red cell aplasia that begins after treatment with darbepoetin alfa or other erythropoietin protein drugs	• Poorly controlled hypertension
	• Serious allergic reactions to darbepoetin alfa	• Poorly controlled hypertension
Other	• Boxed warning for increased risk of death, myocardial infarction, stroke, venous thromboembolism, thrombosis of vascular access, and tumor progression or recurrence.	Effect on tumor growth listed under “Special warnings and precautions for use” (section 4.4, full Summary of Product Characteristics).
	• REMS program in place (ESA APPRISE)^52^	

Refer to the full U.S. prescribing information or summary of product characteristics for complete information. APPRISE, assisting providers and cancer patients with risk information for the safe use of ESAs; CIA, chemotherapy-induced anemia; ESA, erythropoiesis-stimulating agent; EU, European Union; REMS, risk evaluation and mitigation strategy.

## Review of ESA Data in Oncology

Although more than 90 studies have been conducted on the use of ESAs in patients with cancer, most were not powered for survival analyses. Moreover, many studies tested ESAs outside of the approved label (e.g., higher trigger and/or target hemoglobin levels, with radiotherapy alone, or without chemotherapy). Because of the extent of the available data on ESAs, it might be difficult for clinicians to review all the data related to the risk/benefit balance. For this reason, meta-analyses have been conducted as part of PV efforts. By summarizing data from a large number of studies, meta-analyses of large pools of patients increase the statistical power of an analysis to identify potential safety signals that might not be evident in smaller studies 30. Several meta-analyses summarize the risks and benefits of ESAs in patients with various tumor types 31–37 and in the subsets of patients with lung cancer 38 and lymphoproliferative malignancies 39.

In both of the tumor-specific meta-analyses, the use of ESAs was associated with a lower incidence of red blood cell transfusions 31–36,38,39. Reduction in fatigue and improvement of quality of life were also associated with ESA use in the meta-analyses by Grant, Tonelli, Tonia, and Vansteenkiste 32,35,37,38. However, the previously observed increase in the risk of thromboembolic events was confirmed 31,32,34,35,38,39. When the meta-analyses included only patients with CIA, the currently approved indication, no significant effects on mortality 31,33–36,38,39 or disease progression 34,38,39 were seen. However, only meta-analyses with large patient numbers 33,34,37 may have been powered to detect significant mortality differences.

Although meta-analyses have increased statistical power to identify potential safety signals, they may provide only broad, general answers on global clinical questions and may have limitations resulting from the aggregation of heterogeneous data 30. Meta-analyses often incorporate populations with various tumor types, disease stages, and treatment regimens. Study design characteristics such as endpoints and statistical methods may also differ. As a result, it may be difficult for physicians to apply the general results of meta-analysis to treatment decisions for individual patients in daily clinical practice.

Results from individual studies of patients with specific tumor types have provided more focused data. Data from the postmarketing commitment studies in breast, cervical, and lung cancer and NHL showed no significant difference between ESAs and control groups for overall and progression-free survival 17,18,20–22,40. One other large randomized double-blind study in Hodgkin lymphoma also showed no significant difference in overall and progression-free survival endpoints 41. In all of these studies, as expected, the incidence of transfusions was lower and the incidence of thromboembolic events was higher in the ESA groups (individual studies published since 2011 are shown in Table 3) 17,18,20–22,40,41.

**Table 3:** Key publications: ESA use in oncology

Publication	Cancer type	Cancer and anemia treatments	Number of patients	Overall survival	Progression-free survival	RBC transfusions	Thromboembolic adverse events
Studies with reported safety signals in anemia^1^							
Leyland-Jones 6,10 BEST Study	Metastatic breast cancer	First-line chemotherapy; epoetin alfa or placebo	939	12 months: ESA: 70% Control: 76% HR 1.37 95% CI 1.07–1.75 *P* = 0.012 [6]	Disease progression at 12 months: ESA: 41% Control: 43% HR 1.00; *P* = 98 10	ESA: 10% Control: 14% *P* < 0.06 [10]	ESA: 16% Control: 14%
Untch et al. 6,19,40 PREPARE Study	Primary breast cancer	Neoadjuvant chemotherapy; darbepoetin alfa or control	733	3 years: ESA: 86% Control: 90% HR 1.42 95% CI 0.93–2.18 6	3 years: ESA: 72% Control: 78% HR 1.33 95% CI 0.99–1.79 [6]	ESA: 0.3% Control: 0% 40	ESA: 6.3% Control: 4.3% 40
Thomas et al. 13 GOG-191 Study	Cervical cancer	Chemoradiotherapy, epoetin alfa versus control	114	3 years: ESA: 61% Control: 71% HR 1.28 95% CI 0.68–2.42 6	3 years: ESA: 59% Control: 62% HR 1.06 95% CI 0.58–1.91 6	ESA: 59.6% Control: 55.8% 13	ESA: 19% Control: 9% 6
Henke et al. 11	Head and neck cancer	Radiotherapy; epoetin alfa versus placebo	351	HR 1.39 95% CI 1.05–1.84 *P* = 0.02 6	ESA: 406 days Control: 745 days HR 1.62 95% CI 1.22–2.14 *P* = 0.0008 6	Not reported	Vascular disorders: ESA: 11% Control: 5% 11
Overgaard et al. 28 DAHANCA 10 Study	Head and neck cancer	Radiotherapy; darbepoetin alfa versus control	522; 484 analyzed	RR 1.28 95% CI 0.98–1.68 *P* = 0.08 6	5 years, locoregional control RR 1.44 95% CI 1.06–1.96 *P* = 0.02 6	Not reported	Serious cardiovascular events: ESA: 3% Control: 1% 28
Hedenus et al. 6,25,39 Study 20000161	Lymphoproliferative malignancies	Chemotherapy; darbepoetin alfa or placebo	344	29 months: HR 1.36 95% CI 1.02–1.82 [6]	Disease progression or death at 11 months ESA: 47% Control: 45%^25^	ESA: 31% Control: 48% *P* < 0.001 [25]	ESA: 6% Control: 4% 39
Wright et al. 6,27	NSCLC	No systemic treatment or radiation; epoetin alfa versus placebo	70	ESA: 63 days Control: 129 days HR 1.84; *P* = 0.04 [6]	Not reported	ESA: 15% Control: 27% 27	ESA: 3% Control: 5% [27]
Smith et al. 6,53	Nonmyeloid malignancies	Neither chemotherapy nor radiotherapy; darbepoetin alfa versus placebo	989	ESA: 8 months Control: 10.8 months HR 1.30 95% CI 1.07–1.57 6,21	Not reported	ESA: 19% Control: 24% 53	ESA: 2.3% Control: 1.5% 53
Meta-analyses published since 2009							
Ludwig et al. 31	Various tumor types	Chemotherapy; darbepoetin alfa or placebo	2122	HR 0.97 95% CI 0.85–1.10	HR 0.83 95% CI 0.84–1.04	Not reported	ESA: 8% Control: 5% HR 1.57 95% CI 1.10–2.26
Tonelli et al. 32	Various tumor types	Chemotherapy, surgery, radiotherapy, or no cancer treatment; any ESA or control	12,006	All patients: RR 1.15 95% CI 1.03–1.29 Patients receiving chemotherapy: RR 1.04 95% CI 0.86–1.26	Not reported	RR 0.64 95% CI 0.56–0.73	Cardiovascular events: RR 1.12 95% CI 0.83–1.50
Bohlius et al. 54 (Cochrane analysis)	Various tumor types	Chemotherapy, radiotherapy, or no cancer treatment; any ESA with or without control	13,933	All patients: cHR 1.06 95% CI 1.00–1.12 *P* = 0.046 Patients receiving chemotherapy: cHR 1.04 95% CI 0.97–1.11	Not reported	Not reported	Not reported
Glaspy et al. 34	Various tumor types	Chemotherapy, radiotherapy, or no cancer treatment; any ESA with or without control	All patients: 15,323 Patients receiving chemotherapy: 12,108	All patients: OR 1.06 95% CI 0.97–1.15 Patients receiving chemotherapy: OR 1.03 95% CI 0.93–1.13	All patients: OR 1.01 95% CI 0.90–1.14 Patients receiving chemotherapy: OR 0.94 95% CI 0.85–1.06	Not reported	OR 1.48 95% CI 1.28–1.72
Tonia et al. 35 (Cochrane analysis)	Various tumor types	Chemotherapy subset analyses; any ESA with or without control	All patients: 20,102 Patients receiving chemotherapy: 13,800	All patients: HR 1.05 95% CI 1.0–1.11 Patients receiving chemotherapy: HR 1.04 95% CI 0.98–1.11	Not reported	All patients: RR 0.65 95% CI 0.62–0.68 Patients receiving chemotherapy: RR 0.64 95% CI 0.61–0.67	All patients: RR 1.52 95% CI 1.34–1.74 Patients receiving chemotherapy: RR 1.48 95% CI 1.27–1.73
Vansteenkiste et al. 38	Lung cancer (NSCLC and SCLC)	Chemotherapy, radiotherapy, combination, or none; any ESA or control	2342	Study-level analysis: OR 0.87 95% CI 0.69–1.09 Patient-level analysis: HR 0.90 95% CI 0.78–1.03	Study-level analysis: OR 0.84 95% CI 0.65–1.09 Patient-level analysis: HR 0.92 95% CI 0.81–1.06	Week 5 to end of study Study-level analysis OR 0.34 95% CI 0.29–0.41 Patient-level analysis: ESA 19% Control: 43%	ESA: 10.5% Control: 7.2%
Hedenus et al. 39	Lymphoproliferative malignancies	Chemotherapy; any ESA or control	2866	OR 1.05 95% CI 0.81–1.34	OR 1.02 95% CI 0.81–1.30	Individual study data: ESA: 19–63% Control: 28–82%	Individual study data: ESA: 3–9% Control: 0–4%
Grant et al. 37	Various tumor types	Chemotherapy, radiotherapy, combination, or none; any ESA or control	14,278	RR 1.04 95% CI 0.99–1.10	Not reported	RR 0.58 95% CI 0.53–0.64	RR 1.51 95% CI 1.30–1.74
Individual studies published since 2011							
Blohmer et al. 20	High-risk cervical cancer	Chemotherapy; epoetin alfa or control	257	HR 0.88 95% CI 0.51–1.50	HR 0.66 95% CI 0.39–1.12	ESA: 10.7% Control: 29.6% *P* < 0.001	ESA: 1.6% Control: 2.4%
Möbus et al. 17	Node-positive breast cancer	Chemotherapy; epoetin alfa or control	1284	5 years: HR 0.97 95% CI 0.67–1.41 *P* = 0.89	5 years: HR 1.03 95% CI 0.77–1.37 *P* = 0.86	ESAs: 12.8% Control: 28.1% *P* < 0.0001	ESA: 7% Control: 3%
Delarue et al. 22,2 LNH03-6B Study	Diffuse large B-cell lymphoma	Chemotherapy; darbepoetin alfa or standard of care	600	HR 0.81 95% CI 0.60–1.09 *P* = 0.16	3 years: Progression-free survival: HR 0.77 95% CI 0.59–0.99 *P* = 0.04 Disease-free survival: HR 0.65 95% CI 0.45–0.92 *P* = 0.02	Not reported	ESA: 13% Control: 6%
Ongoing safety studies							
Study EPO-ANE-3010 42	Metastatic breast cancer	Chemotherapy; epoetin alfa or standard of care	Estimate 2100	No data yet reported; estimated completion date 2017			
Study 20070782 43	Metastatic NSCLC	Chemotherapy; darbepoetin alfa or placebo	Estimate 3000	No data yet reported; estimated completion date 2019			

Control refers to placebo or standard of care as reported in each study. A hazard ratio, rate ratio, or odds ratio less than 1 favors ESAs; a value greater than 1 favors control. ESA, erythropoiesis-stimulating agent; HR, hazard ratio; CI, confidence interval; OR, odds ratio; RR, rate ratio; cHR, combined hazard ratio; NSCLC, non–small-cell lung cancer; SCLC, small-cell lung cancer.

1 For studies cited in product labels, data from the label were used when available; otherwise, data from publications were used as cited.

2 In the Delarue study, 40% of patients in the control arm received ESAs and this may limit the ability to detect differences between the experimental and the control arm. Results reported are for treatment groups as randomized for consistency with other results presented here.

## Ongoing Safety Studies With ESA for Chemotherapy-Induced Anemia

**Figure 3:**
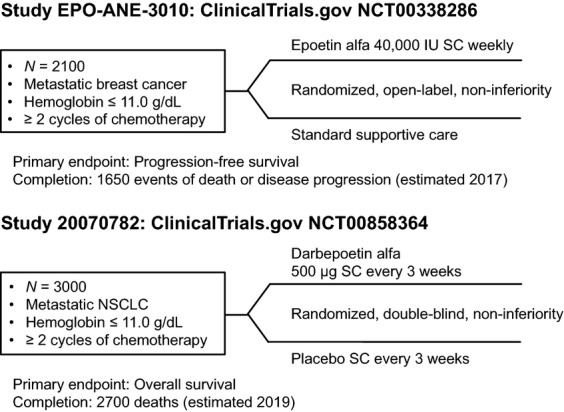
Ongoing safety studies in metastatic breast cancer and non–small-cell lung cancer 42,43. NSCLC, non–small-cell lung cancer; SC, subcutaneous.

As part of ongoing PV efforts to characterize the safety profile of ESAs, two large randomized, multicenter studies are ongoing and enrolling patients globally (Fig.3). These studies were specifically designed in collaboration with regulatory agencies to evaluate if the use of ESA *as indicated* has an adverse effect on survival in patients with CIA. Both studies have a noninferiority design and are adequately powered for survival endpoints. EPO-ANE-3010 42 is a randomized, open-label study of weekly epoetin alfa plus standard supportive care (red blood cell transfusions) versus standard supportive care alone in approximately 2100 anemic patients with metastatic breast cancer receiving chemotherapy. The study's primary endpoint is progression-free survival. Secondary endpoints include overall survival, time to tumor progression, and overall response rate. Results are expected in 2017. Study 20070782 43 is a randomized, double-blind, placebo-controlled study of darbepoetin alfa every 3 weeks versus placebo in approximately 3000 anemic patients with metastatic non–small-cell lung cancer (NSCLC) receiving chemotherapy. A total of 2700 deaths are required to exclude a hazard ratio (darbepoetin alfa:placebo) of 1.15 with a one-sided significance level of 0.025 (the study is powered at over 90% if the true hazard ratio is 1.00). The primary endpoint is overall survival; secondary endpoints include progression-free survival, objective tumor response, and incidence of thromboembolic adverse events. Results are expected in 2019.

## ESA for Chemotherapy-Induced Anemia: Guidelines

Guidelines are regularly updated by several organizations involved in the care of patients with cancer to reflect current evidence 44–47. These guidelines may be useful in evaluating treatment options. Table 4 summarizes current U.S. and European guidelines for the treatment of anemia in patients with cancer.

**Table 4:** Use of ESAs in CIA: published guidelines as of 2013

Guidelines	National Cancer Care Network (NCCN) 45	American Society of Clinical Oncology (ASCO) and American Society of Hematology (ASH) (joint guidelines) 46	European Society for Medical Oncology (ESMO) 47	European Organization for Research and Treatment of Cancer (EORTC) 44
Hemoglobin level for diagnosis of anemia	<10 g/dL	<10 g/dL	≤10 g/dL	9.0–11.0 g/dL based on anemia symptoms 11.0–11.9 g/dL for selected patients to prevent further decline
Target hemoglobin level	Lowest level that avoids transfusion	Lowest level that avoids transfusion	Not exceed 12 g/dL	About 12 g/dL
Indication/Initiation	• Only to be administered under REMS program (ESA APPRISE) as indicated by U.S. prescribing information	• Patients undergoing myelosuppressive chemotherapy to decrease transfusions	• Treatment of symptomatic CIA in nonmyeloid malignancies.	• Patients receiving chemotherapy or radiochemotherapy
	• For anemia with myelosup-pressive chemotherapy without other identifiable cause of anemia, ESA may be considered	• Not for curative intent	• Use with caution with chemotherapy with curative intent	• Not for prophylaxis
	• In anemic patients undergoing palliative treatment, ESA may be considered			
	• Should not be used if the anticipated outcome is cure			
Iron supplementation	Patients receiving ESA developing functional iron deficiency will likely benefit from IV iron	Insufficient evidence to consider the use of IV iron as standard of care	IV iron leads to higher Hb increment in comparison with oral or no iron substitution	There is evidence of better response to ESAs with IV iron

Hb, hemoglobin; IV, intravenous; RBC, red blood cell; REMS, Risk Evaluation and Mitigation Strategy.

## Biosimilars and PV

Since 2007, biosimilars to epoetin alfa have become available in Europe, including three brands of epoetin alfa: Hexal (Hexal, Holzkirchen, Germany), Abseamed (Medice Arzneimittel Pütter, Iserlohn Germany), and Binocrit (Sandoz, Kundl, Austria), and two brands of epoetin zeta: Retacrit (Hospira, Warwickshire, UK) and Silapo (Stada, Bad Vilbel, Germany) 48. Biosimilars are subject to regulatory reviews different from those implemented for either innovator or generic drugs. The importance of PV activities has recently been underscored by reports of immunogenicity with biosimilars of epoetin alfa administered subcutaneously in chronic kidney disease 49. Aggregation of proteins caused by devices in use (e.g., tungsten exposure in prefilled syringes 50), formulations (e.g., replacement of the stabilizer human-sourced albumin with polysorbate 80 51), or inappropriate handling discovered in these situations strongly supports the need for robust PV programs for biosimilars including long-term follow-up of patients included in clinical trials.

## Summary

PV is the science and activities relating to the detection, assessment, understanding, and prevention of adverse effects related to licensed medical products. ESAs are used to treat anemia in patients with cancer receiving concomitant myelosuppressive chemotherapy; the history of ESA use in the United States provides an example of PV and its application to a medical product. In the early postapproval period, results of studies performed outside of the approved indications suggested that ESAs were associated with an increased risk of adverse outcomes. PV efforts including meta-analyses and individual studies helped to characterize this risk. Clinical studies have consistently confirmed that ESAs increase hemoglobin and reduce the need for transfusions, but also that they increase the risk of thromboembolic adverse events. No significant effect of ESAs on survival or disease progression has been shown in studies within the approved indication of patients with CIA. Two large randomized trials in breast cancer and NSCLC are ongoing and enrolling patients globally; results of these studies in the next 5–7 years will further clarify the risks and benefits of ESAs when used in accordance with product labeling.

## Conclusions

Robust PV programs are an essential and continuing effort; new data are continuously being evaluated to ensure that drugs are safe for their indicated uses. Ongoing studies are expected to shed additional light on the effects of ESAs on survival and disease progression in patients with chemotherapy-induced anemia.
